# RETRACTION: Neurodegenerative Shielding by Curcumin and Its Derivatives on Brain Lesions Induced by 6‐OHDA Model of Parkinson’s Disease in Albino Wistar Rats

**DOI:** 10.1155/cpn/9839183

**Published:** 2026-06-04

**Authors:** Cardiovascular Psychiatry and Neurology

RETRACTION: S. S. Agrawal, S. Gullaiya, V. Dubey, et al., “Neurodegenerative Shielding by Curcumin and Its Derivatives on Brain Lesions Induced by 6‐OHDA Model of Parkinson’s Disease in Albino Wistar Rats,” *Cardiovascular Psychiatry and Neurology* 2012, no. 1 (2012): 942981, https://doi.org/10.1155/2012/942981.

The above article, published online on 15 August 2012 in Wiley Online Library (wileyonlinelibrary.com), has been retracted by John Wiley & Sons Ltd. The retraction has been agreed due to multiple inconsistencies in Figure 2, initially identified on PubPeer [1]. All five panels in Figure 2 appear to contain sections which overlap with each other, and there are also unexpectedly similar regions within the panels.

The results and conclusions are therefore considered unreliable.

The authors did not respond when notified of the retraction.
